# Primary Esophageal Melanoma with Aberrant CD56 Expression: A Potential Diagnostic Pitfall

**DOI:** 10.1155/2017/9052637

**Published:** 2017-11-05

**Authors:** Hani Katerji, John M. Childs, Laura E. Bratton, Christian G. Peyre, Aaron R. Huber

**Affiliations:** ^1^Department of Pathology and Laboratory Medicine, University of Rochester, Rochester, NY, USA; ^2^Walter Reed National Military Medical Center, Department of Dermatopathology, Bethesda, MD, USA; ^3^Ochsner Clinic, Department of Pathology, New Orleans, LA, USA; ^4^Department of Surgery, Division of Thoracic and Foregut Surgery, University of Rochester, Rochester, NY, USA

## Abstract

Primary esophageal malignant melanoma (MM) is rare and extremely aggressive. For pathologists, it can be challenging to diagnose and differentiate from other poorly differentiated malignant neoplasms in the esophagus. Complicating this fact, MM can have divergent differentiation and express nonmelanocytic immunohistochemical markers including epithelial markers (cytokeratins) and rarely neuroendocrine markers. Lack of awareness of this fact by a pathologist can lead to an erroneous diagnosis and delay treatment for an already aggressive disease. Herein, we report a case of primary esophageal malignant melanoma with aberrant CD56 expression without accompanying synaptophysin or chromogranin expression.

## 1. Background

Malignant melanoma (MM) is the sixth most common malignancy in United States, continuously increasing in incidence [1]. The diagnosis of MM can be challenging due to the heterogeneity and histologic diversity of this neoplasm. This may be even more difficult when MM occurs at unusual locations and/or has aberrant expression of nonmelanoma antigens [2, 3]. We present a case of esophageal MM that demonstrated aberrant expression of CD56 while lacking other neuroendocrine markers including synaptophysin and chromogranin.

## 2. Case Summary

An 82-year-old woman presented with a recent history of dysphagia and heartburn. She underwent upper endoscopy which demonstrated a fungating mass extending 10 cm in length and protruding into and filling the lumen. The edges were necrotic and biopsies were obtained from the base of the lesion.

**Figure 1:**
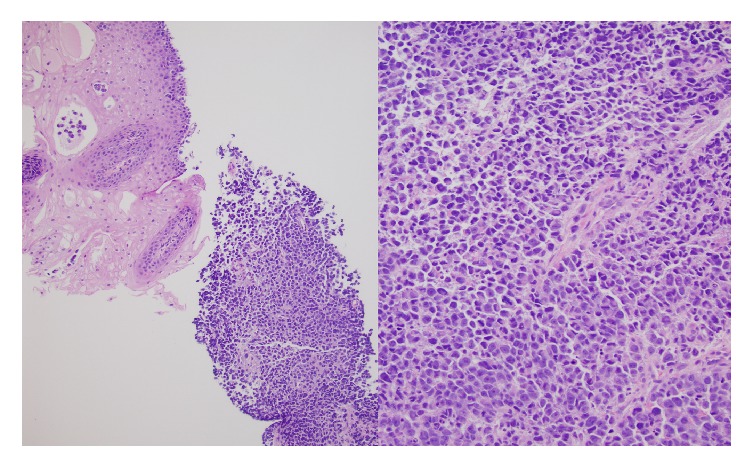
H+E stain showing the tumor cells replacing the squamous mucosa (left side, 10x). Discohesive malignant tumor cells growing in sheets (H&E, right, 20x).

**Figure 2:**
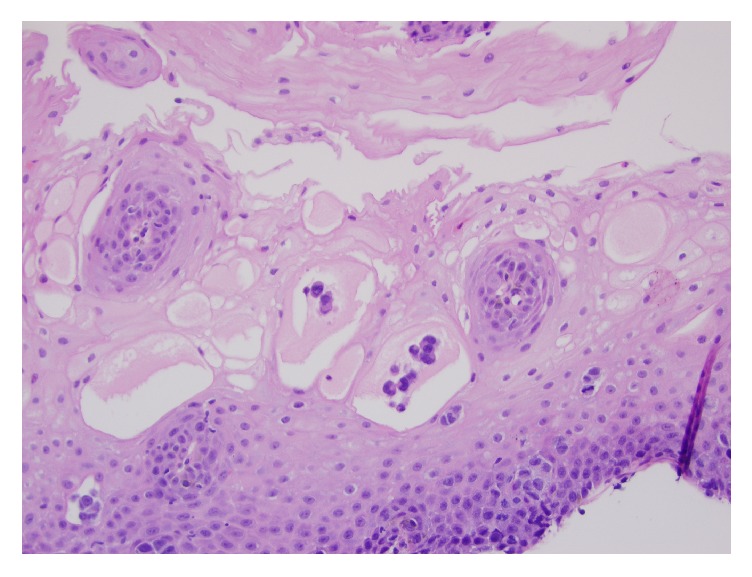
Pagetoid involvement of the squamous mucosa by the neoplastic cells (H&E, 20x).

Histology showed discohesive, small to medium sized cells, arranged primarily in solid nests, with focal necrosis (Figure 1). Cytologically, the cells showed scant to moderate amounts of cytoplasm and eccentric, irregular nuclei with prominent nucleoli. There was focal pagetoid involvement of the overlying squamous mucosa (Figure 2).

**Figure 3:**
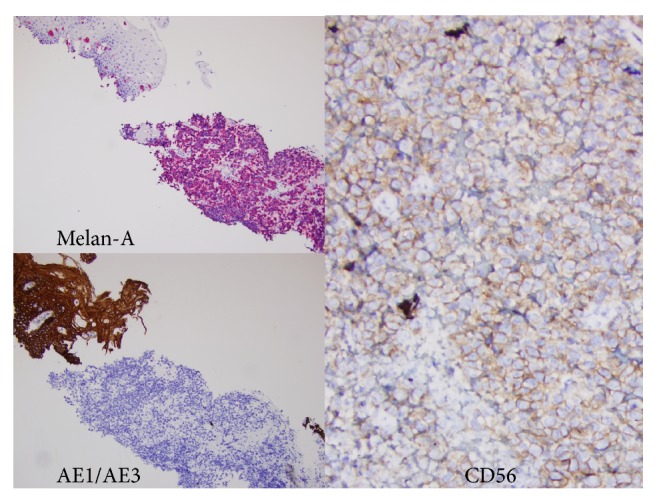
The malignant cells are positive for Melan-A and CD56 with a negative cytokeratin AE1/AE3.

Immunohistochemical stains were performed and the neoplastic cells were negative for cytokeratin AE1/AE3, cytokeratin cocktail, synaptophysin, chromogranin, p63, thyroid transcription factor 1 (TTF-1), leukocyte common antigen, and other lymphoid markers while they were diffusely positive for CD56. This immunophenotype excluded the possibility of squamous cell carcinoma. Then, stains for malignant melanoma were performed and demonstrated that the neoplastic cells were focally positive for S-100 protein while other MM markers including Melan-A and SOX10 were diffusely positive (Figure 3).

Based on the morphology and immunohistochemical phenotype, and the absence of a primary tumor elsewhere, a diagnosis of primary esophageal melanoma with aberrant expression of CD56 was rendered.

Molecular testing did not reveal a V600E or V600K mutation in the* BRAF* oncogene or in the* C-kit* oncogene.

**Figure 4:**
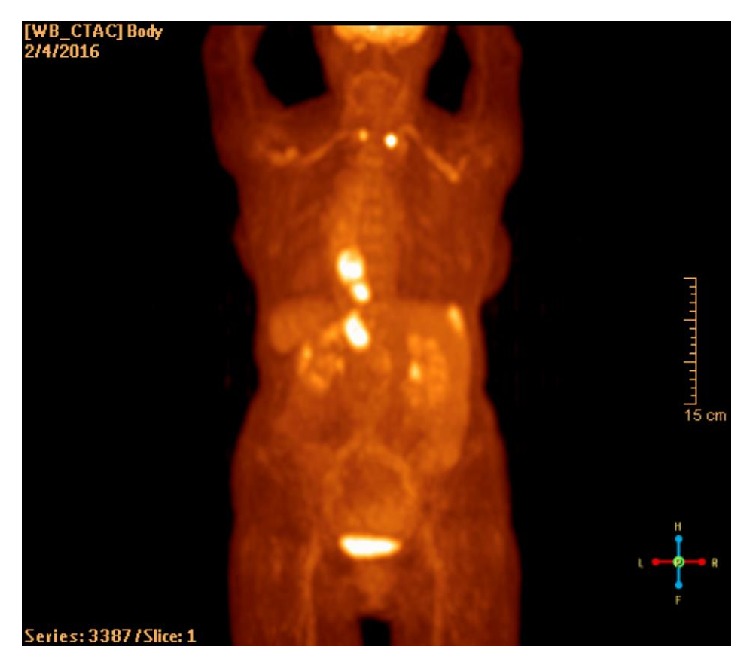
PET scan demonstrating a hypermetabolic mass in the distal esophagus. Also, a hypermetabolic liver lesion and an enlarged gastroesophageal lymph node are seen. This PET scan incidentally revealed bilateral hypermetabolic thyroid lesions.

A positron emission tomography (PET) scan was performed and highlighted the mass in the distal esophagus. It also revealed a 2 cm hypermetabolic liver lesion and hypermetabolic gastroesophageal lymph node (Figure 4). This PET scan also incidentally revealed bilateral hypermetabolic thyroid lesions. Fine needle aspiration of the thyroid nodules revealed papillary thyroid carcinoma.

Chemotherapy was initiated with pembrolizumab, a monoclonal antibody that is given for metastatic or unresectable melanoma, but the patient's disease progressed with more extensive hepatic involvement by metastatic disease, and she died shortly thereafter.

## 3. Discussion

Cutaneous malignant melanoma, a malignant tumor of melanocytes, is most prevalent in patients over the age of 30 years. It can be sporadic or inherited, with the latter seen in a minority of patients. The melanocytes are derived from the neural crest and are widely distributed throughout all cutaneous and most mucosal surfaces. Even though the most common sites of primary MM are the skin and choroidal layer of the eye, it can occur in other locations including the central nervous system, gastrointestinal tract, and genitourinary tract [2].

The vast majority of MM cases in the gastrointestinal tract are metastatic, typically from a cutaneous primary melanoma; however, there is evidence that melanoma can arise de novo from within certain areas of the gastrointestinal system, including the esophagus, small bowel, colon, rectum, and anus [3].

Primary esophageal melanoma is a very rare malignancy, comprising less than 1% of esophageal malignant tumors [4]. The etiology by which these tumors arise is not well understood, but it may be related to ectopic melanocyte precursors that migrate down the upper two-thirds of the esophagus during development [5]. The diagnosis of primary esophageal MM can be challenging but pathologists should entertain this diagnosis when confronted with a biopsy of a large or polypoid luminal mass. Strict pathologic criteria must be used when diagnosing a primary esophageal melanoma. The major criterion for the diagnosis of primary esophageal MM is the presence of a junctional component in the overlying squamous mucosa [6]. However, when the tumors are bulky and there is overlying ulceration, the junctional component may not be identifiable [6]. Microscopically, MM is comprised of neoplastic cells with or without melanin pigment (amelanotic melanoma), arranged in nests or sheets [6]. Cytologically, the cells are spindled to epithelioid with moderate amounts of cytoplasm and large, vesicular nuclei with prominent nucleoli and may show pagetoid involvement of the squamous mucosa [6]. But, as our case suggests, this tumor can cause diagnostic difficulty particularly on a small biopsy; thus, immunohistochemical staining is extremely helpful in reaching a correct diagnosis. In fact about 25% of cases of primary esophageal MM are incorrectly diagnosed on biopsy and the correct diagnosis is only made at resection [6]. A melanocytic immunophenotype with multiple positive markers should lead a pathologist to the correct diagnosis but it is important to recognize that MM may aberrantly express cytokeratins [7, 8], rarely have neuroendocrine differentiation [8–11], or may show other divergent types of differentiation [7, 8, 11].

In a review, Banerjee and Eyden [7] demonstrated that MM, like other tumors, may show divergent differentiation. The types of differentiation reported in malignant melanoma include fibroblastic/myofibroblastic, schwannian and perineurial, smooth muscle, rhabdomyosarcomatous, osteocartilaginous, ganglionic and ganglioneuroblastic, neuroendocrine, and probable epithelial. MM with neuroendocrine differentiation is an extremely rare phenomenon, and when it occurs it can lead to diagnostic uncertainty. Romano et al. [8] have also shown that a significant number of MM cases may demonstrate aberrant expression of intermediate filaments and/or synaptophysin. They found anomalous intermediate filament expression in 48% of cases and synaptophysin in 28%. Eyden et al. [9] described three cases of MM with neuroendocrine differentiation in which the neoplastic cells were positive for melanocytic and neuroendocrine markers—chromogranin, synaptophysin, neurofilament protein, and HMB-45. Two of the three cases were stained for CD56, and they were both positive [5]. Riddle and Bui [11] also reported a case of metastatic malignant melanoma that expressed chromogranin, CD56, and cytokeratin AE1/AE3 but was S-100 protein negative in a fine needle aspiration specimen. In the resection specimen, S-100 protein was positive. They pointed out that aberrant expression of cytokeratin and neuroendocrine markers and intratumoral heterogeneity in the expression of certain antigens (i.e., S-100 protein) are important potential pitfalls in the diagnosis of MM.

The histologic differential diagnosis of malignant melanoma of the esophagus is broad and includes lymphoma, poorly differentiated carcinoma (either squamous or adenocarcinoma), a high-grade neuroendocrine neoplasm, and possibly a metastasis or direct extension of a primary tumor elsewhere. This differential can be sorted out with a panel of immunohistochemical stains. S-100 protein is the most sensitive but least specific marker for MM [12]. Melanoma antigen recognized by T cells (MART-1), microphthalmia-associated transcription factor (MITF), and human melanoma black-45 (HMB-45), melan-A, and tyrosinase are common markers that have increased specificity for MM [9, 12]. In this case of primary esophageal melanoma, the tumor cells expressed all of the melanocytic markers including S-100 protein, melan-A, and SOX-10. Neural cell adhesion molecule (CD56) is normally expressed on neurons, glial tissue, skeletal muscle, and natural killer cells [13]. CD56 lacks the specificity of other neuroendocrine markers [13]. An unexpected finding was the positivity of the tumor cells for CD56 while they were negative for the other neuroendocrine markers. We cannot consider this true neuroendocrine differentiation since CD56 is a nonspecific neuroendocrine marker and the tumor lacked expression of other, more specific, neuroendocrine markers such as chromogranin and synaptophysin. However, this aberrant expression of CD56 should remind pathologists about the heterogeneity of this tumor, and the possibility that MM may aberrantly express markers of divergent lineage [7, 8, 10, 11, 14].

In summary, esophageal MM, either primary or metastatic, is extremely rare and aggressive. The diagnosis requires a high index of suspicion and melanoma should be included in the histologic differential diagnosis of poorly differentiated neoplasms in the esophagus [3]. The potential misdiagnosis as a neuroendocrine carcinoma/tumor is a diagnostic pitfall particularly if aberrant neuroendocrine marker expression is present. Pathologists should be aware of the possibility of neuroendocrine marker expression in melanoma to avoid this potential diagnostic pitfall [7, 8, 10, 11, 14].
